# Supplementary material for: Candida auris undergoes adhesin-dependent and -independent cellular aggregation
Source: PLoS Pathog. 2024 Mar 11;20(3):e1012076. doi: 10.1371/journal.ppat.1012076 (PMC10957086; doi:10.1371/journal.ppat.1012076)
Supplement: S1 Table — (DOCX) [file ppat.1012076.s001.docx]

**Table S1.** MIC_90_ of Caspofungin (CSP) and Micafungin (MFG), MIC_90_ as identified by E-test strips

| Strain | CSP (mg/L) MIC_90_ | MFG (mg/L) MIC_90_ |
| --- | --- | --- |
| UACa11 | 12 | 0.094* |
| UACa25 | 1 | 0.094* |
| UACa10 | 0.175 | 0.094 |
| UACa20 | 0.38 | 0.064 |

*Actual MIC with no growth above indicated concentration.
