# Supplementary material for: Candida auris undergoes adhesin-dependent and -independent cellular aggregation
Source: PLoS Pathog. 2024 Mar 11;20(3):e1012076. doi: 10.1371/journal.ppat.1012076 (PMC10957086; doi:10.1371/journal.ppat.1012076)
Supplement: S2 Table — (DOCX) [file ppat.1012076.s002.docx]

**Table S2.** Genes upregulated in both *C. auris* UACa20 and UACa11 during growth in SabDex but with a greater than log2FC difference between strains

| **Gene** | **UACa20 log2FC** | **UACa11 log2FC** | **Potential homolog in *C. albicans*** |
| --- | --- | --- | --- |
| CJI97_004514 | 9.49 | 7.42 | *THI13* |
| CJI97_003172 | 7.55 | 9.66 | *THI4* |
| CJI97_002670 | 4.24 | 2.07 | C3_00990C_A |
| CJI97_000357 | 4.19 | 1.14 | - |
| CJI97_000368 | 3.64 | 1.61 | C3_03440C_A |
| CJI97_001224 | 2.11 | 4.55 | *RBT7* |
| CJI97_001401 | 1.95 | 4.08 | *THI6* |
| CJI97_004176 | 1.66 | 3.85 | *MDR1* |
