# Supplementary material for: Candida auris undergoes adhesin-dependent and -independent cellular aggregation
Source: PLoS Pathog. 2024 Mar 11;20(3):e1012076. doi: 10.1371/journal.ppat.1012076 (PMC10957086; doi:10.1371/journal.ppat.1012076)
Supplement: S3 Table — (DOCX) [file ppat.1012076.s003.docx]

**Table S3.** DEGs expressed in opposite direction between strains UACa20 and UACa11

| **Gene** | **UACa20 log2FC** | **UACa11 log2FC** | **Potential homolog in *C. albicans*** |
| --- | --- | --- | --- |
| CJI97_001729 | 3.75 | -1.45 | CR_01630C_A |
| CJI97_004216 | 1.67 | -1.01 | *HBR1* |
| CJI97_000418 | 1.53 | -1.23 | *CFL4* |
| CJI97_004611 | 1.53 | -2.70 | *CDA2* |
| CJI97_001311 | 1.52 | -2.84 | C6_02660C_A |
| CJI97_000125 | 1.36 | -1.10 | *TSR1* |
| CJI97_005371 | 1.28 | -2.36 | *SAP5* |
| CJI97_004564 | 1.21 | -1.90 | *SAP8* |
| CJI97_004013 | -1.21 | 1.36 | *PLB4.5* |
| CJI97_001240 | -1.22 | 1.23 | C4_07040W_A |
| CJI97_004556 | -1.78 | 3.55 | *PRD1* |
| CJI97_005598 | -2.42 | 1.37 | C2_06350C_A |
| CJI97_004563 | -2.66 | 1.67 | *DUR3* |
