# Supplementary material for: Candida auris undergoes adhesin-dependent and -independent cellular aggregation
Source: PLoS Pathog. 2024 Mar 11;20(3):e1012076. doi: 10.1371/journal.ppat.1012076 (PMC10957086; doi:10.1371/journal.ppat.1012076)
Supplement: S4 Table — (DOCX) [file ppat.1012076.s004.docx]

**Table S4.** Top 10 upregulated DEGs during growth in SabDex unique to *C. auris* strain UACa11

| **Gene** | **log2FC** | **Potential homolog in *C. albicans*** |
| --- | --- | --- |
| CJI97_002126 | 3.94 | *ALS4* |
| CJI97_004172 | 3.71 | *RBR3* |
| CJI97_002948 | 3.27 | C1_10310W_A |
| CJI97_004914 | 2.90 | *OPT3* |
| CJI97_002178 | 2.88 | *CYB2* |
| CJI97_004170 | 2.87 | - |
| CJI97_004560 | 2.75 | *HYR3* |
| CJI97_004890 | 2.71 | *OPT1* |
| CJI97_004892 | 2.59 | *OPT3* |
| CJI97_005489 | 2.55 | *TRA1* |
