# Supplementary material for: Candida auris undergoes adhesin-dependent and -independent cellular aggregation
Source: PLoS Pathog. 2024 Mar 11;20(3):e1012076. doi: 10.1371/journal.ppat.1012076 (PMC10957086; doi:10.1371/journal.ppat.1012076)
Supplement: S5 Table — (DOCX) [file ppat.1012076.s005.docx]

**Table S5.** Top 10 upregulated DEGs during growth in SabDex unique to *C. auris* strain UACa20

| **Gene** | **log2FC** | **Potential homolog in *C. albicans*** |
| --- | --- | --- |
| CJI97_000210 | 3.70 | C3_02040C_A |
| CJI97_004175 | 3.67 | *ALS4* |
| CJI97_003074 | 3.50 | - |
| CJI97_003045 | 3.29 | *VHT1* |
| CJI97_004212 | 3.04 | *FAD3* |
| CJI97_000710 | 2.90 | C3_04450C_A |
| CJI97_002719 | 2.71 | *AGP2* |
| CJI97_003695 | 2.70 | C2_04080W_A |
| CJI97_003085 | 2.62 | *SRP40* |
| CJI97_000246 | 2.57 | C1_10970W_A |
