# Supplementary material for: Candida auris undergoes adhesin-dependent and -independent cellular aggregation
Source: PLoS Pathog. 2024 Mar 11;20(3):e1012076. doi: 10.1371/journal.ppat.1012076 (PMC10957086; doi:10.1371/journal.ppat.1012076)
Supplement: S6 Table — (DOCX) [file ppat.1012076.s006.docx]

| Strain # | Isolate Name | Clade | Source/Reference | Aggregative ability |
| --- | --- | --- | --- | --- |
| UACa6 | NCPF8980#9 | III | PHE, E. Johnson | Yes |
| UACa10 | NCPF13005#95 | III | PHE, E. Johnson | Yes |
| UACa11 | VPCI479/P/13 | I | [1] | No |
| UACa25 | B11098 | I | CDC [3] | No |
| UACa20 | B11221 | III | CDC [3] | Yes |
| UACa22 | B11244 | IV | CDC [3] | Yes |
| UACa83 | CBS10913T | II | [2] | No |
| UACa177 | *als4112*Δ | III | Derivative of UACa20 | No |
| UACa179 | *als4112*Δ + *ALS4112* | III | Derivative of UACa177 | Yes |
| UACa180 | *als4112*Δ + *ALS4112* | III | Derivative of UACa177 | Yes |
| UACa181 | *als4112*Δ + *ALS4112* | III | Derivative of UACa177 | Yes |
| UACa182 | *als4112*Δ + *ALS4112* | III | Derivative of UACa177 | Yes |
| UACa183 | *als4112*Δ + *ALS4112* | III | Derivative of UACa177 | Yes |
| UACa184 | *als4112*Δ + *ALS4112* | III | Derivative of UACa177 | Yes |

**Table S6.** List of *Candida auris* strains

**Supplementary References**

[1] Sharma C, Kumar N, Meis JF, Pandey R, Chowdhary A. Draft genome sequence of a fluconazole-resistant *Candida auris* strain from a candidemia patient in India. Genome Announcements 2015;3:e00722-15. https://doi.org/10.1128/genomeA.00722-15.

[2] Satoh K, Makimura K, Hasumi Y, Nishiyama Y, Uchida K, Yamaguchi H. *Candida auris* sp. nov., a novel ascomycetous yeast isolated from the external ear canal of an inpatient in a Japanese hospital. Microbiol Immunol 2009;53:41–44. https://doi.org/10.1111/j.1348-0421.2008.00083.x.

[3] Lockhart SR, Etienne KA, Vallabhaneni S, Farooqi J, Chowdhary A, Govender NP, et al. Simultaneous emergence of multidrug-resistant C*andida auris* on 3 continents confirmed by whole-genome sequencing and epidemiological analyses. Clinical Infectious Diseases 2017;64:134–140. https://doi.org/10.1093/cid/ciw691.
