# Supplementary material for: Candida auris undergoes adhesin-dependent and -independent cellular aggregation
Source: PLoS Pathog. 2024 Mar 11;20(3):e1012076. doi: 10.1371/journal.ppat.1012076 (PMC10957086; doi:10.1371/journal.ppat.1012076)
Supplement: S7 Table — (DOCX) [file ppat.1012076.s007.docx]

**Table S7.** Sequencing quality report for transcriptomic data

| **Strain** | UACa20 | UACa20 | UACa11 | UACa11 |
| --- | --- | --- | --- | --- |
| **Condition** | SabDex | RPMI-1640 | SabDex | RPMI-1640 |
|  | (Agg) | (Non-Agg) | (Non-Agg) | (Non-Agg) |
| **Raw Reads** | **29,430,966** | **27,484,339** | **27,470217** | **26,102,812** |
| **Filtered Reads** | **29,013,091** | **26,502,460** | **27,195,036** | **25,870,831** |
| **Percentage (%) of filtered reads** |  |  |  |  |
| aligned to ERCC | 0.54 (0.28) | 0.51 (0.21) | 0.45 (0.17) | 0.47 (0.18) |
| aligned to B11221 genome | 98.76 (0.30) | 98.59 (0.33) | 97.79 (0.22) | 97.25 (0.26) |
| aligned as multimappers | 1.29 (0.04) | 2.35 (0.65) | 1.75 (0.18) | 2.61 (0.66) |
| counted as genes | 88.49 (0.23) | 85.24 (0.89) | 86.48 (0.27) | 84.55 (0.83) |

**Reads are the average of the three biological repeats rounded to the nearest whole number. Percentages of filtered reads aligned are given as means with standard deviation in parentheses.**
