# Supplementary material for: Candida auris undergoes adhesin-dependent and -independent cellular aggregation
Source: PLoS Pathog. 2024 Mar 11;20(3):e1012076. doi: 10.1371/journal.ppat.1012076 (PMC10957086; doi:10.1371/journal.ppat.1012076)
Supplement: S8 Table — (DOCX) [file ppat.1012076.s008.docx]

**Table S8.** Oligonucleotide primer list

| **Primer** | **Sequence (5’-3’)** |
| --- | --- |
| oUA315 | TCGTACGCTGCAGGTCGACGGTCGACACTGGATGGCGG |
| oUA316 | CGCGCCTTAATTAACCCGGGATCAAGCTTGCCTCGTCC |
| oUA989 | CCTTATTGCACCCGTGG |
| oUA990 | CGATACTAACGCCGCCATCCAGTGTCGACAGGAAAGTGGAAATTGTGGG |
| oUA991 | GGCGGGGACGAGGCAAGCTTGATCTATGTCTCACACCAAGG |
| oUA992 | GCATTCAAGATGAGATTATTG |
| oUA993 | GCACACTCGACAAGTCTTAGGC |
| oUA994 | CAACAATTTAATATCATAGGAGCTCACG |
| oUA987 | GGCGAAACTGTCACTGTTGT |
| oUA988 | GGTGGCTCAGTGAAGATCCT |
| oUA995 | CAATAGCTTCAGCATCACCTGG |
| oUA996 | CTTTGATTATCAAAGGTTCTCGCTTAGCC |
| oUA997 | GTCTCGTTGCTTAACTGCTGC |
| oUA998 | GGATACAGTTCTCACATCACATCC |
| oUA999 | CCACCTCCCTCGCCAGAC |
| oUA1000 | GCCAGTCGTGATGTGTGC |
| oUA1025 | GTTTCCTGTCTTAGTTTGCC |
| oUA1026 | CATGGCATTCGTTCATATCC |
| oUA1027 | GGCAAACTAAGACAGGAAACCGAAAGGGCCTCGTGATACG |
| oUA1028 | GGATATGAACGAATGCCATGCCATCCGAACGTTTTTGTGC |
