# Supplementary figures and images for: Candida auris undergoes adhesin-dependent and -independent cellular aggregation
Source: PLoS Pathog. 2024 Mar 11;20(3):e1012076. doi: 10.1371/journal.ppat.1012076 (PMC10957086; doi:10.1371/journal.ppat.1012076)

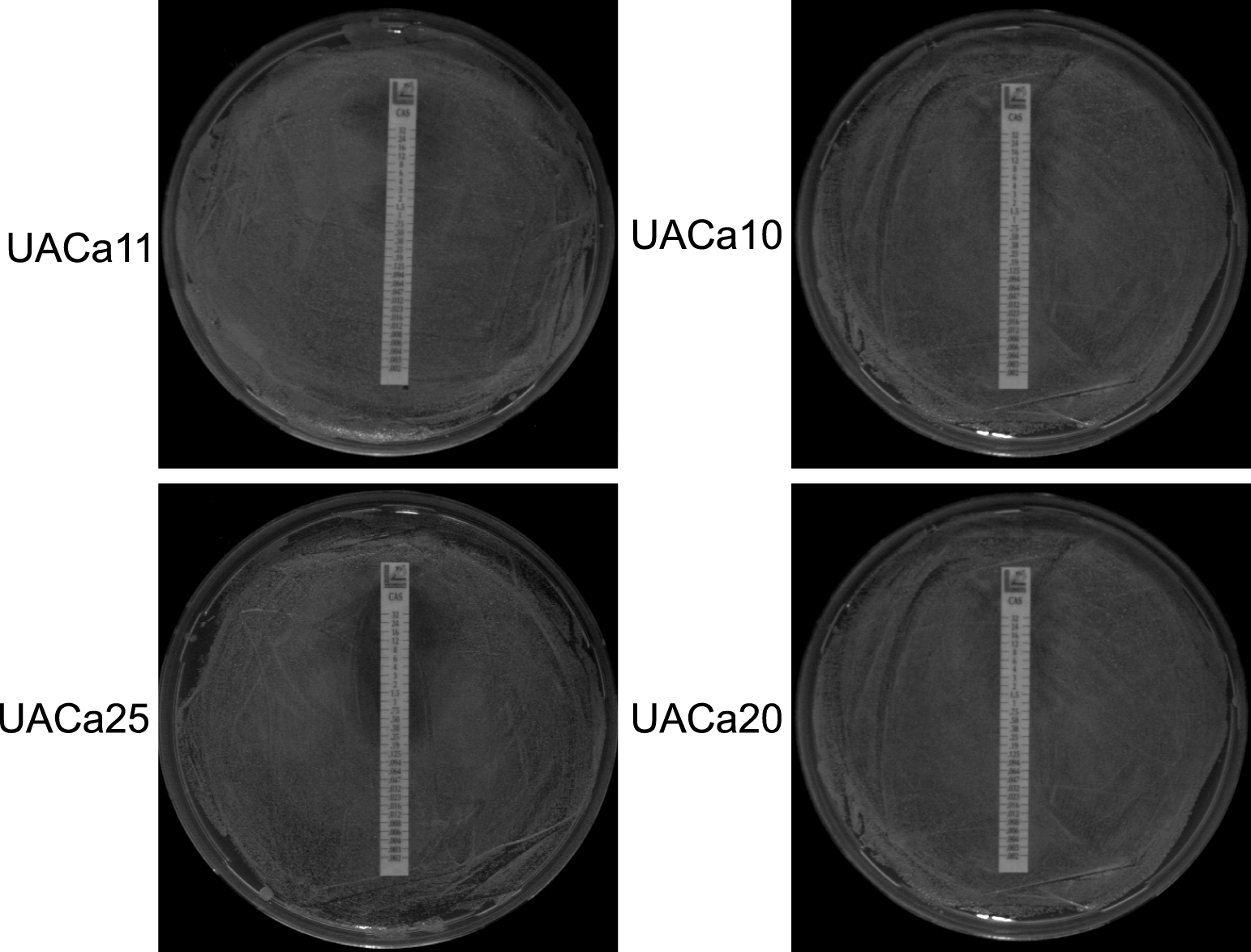

Supplement: S1 Fig — All strains had growth up to 32 mg/L along the E-test strip, MIC90 was determined from where growth was decreased, for UACa11 this was 12 mg/L, for UACa25 1 mg/L, for UACa10 0.175 mg/L, and for UACa20 0.38 mg/L. (TIF) [file ppat.1012076.s009.tif]

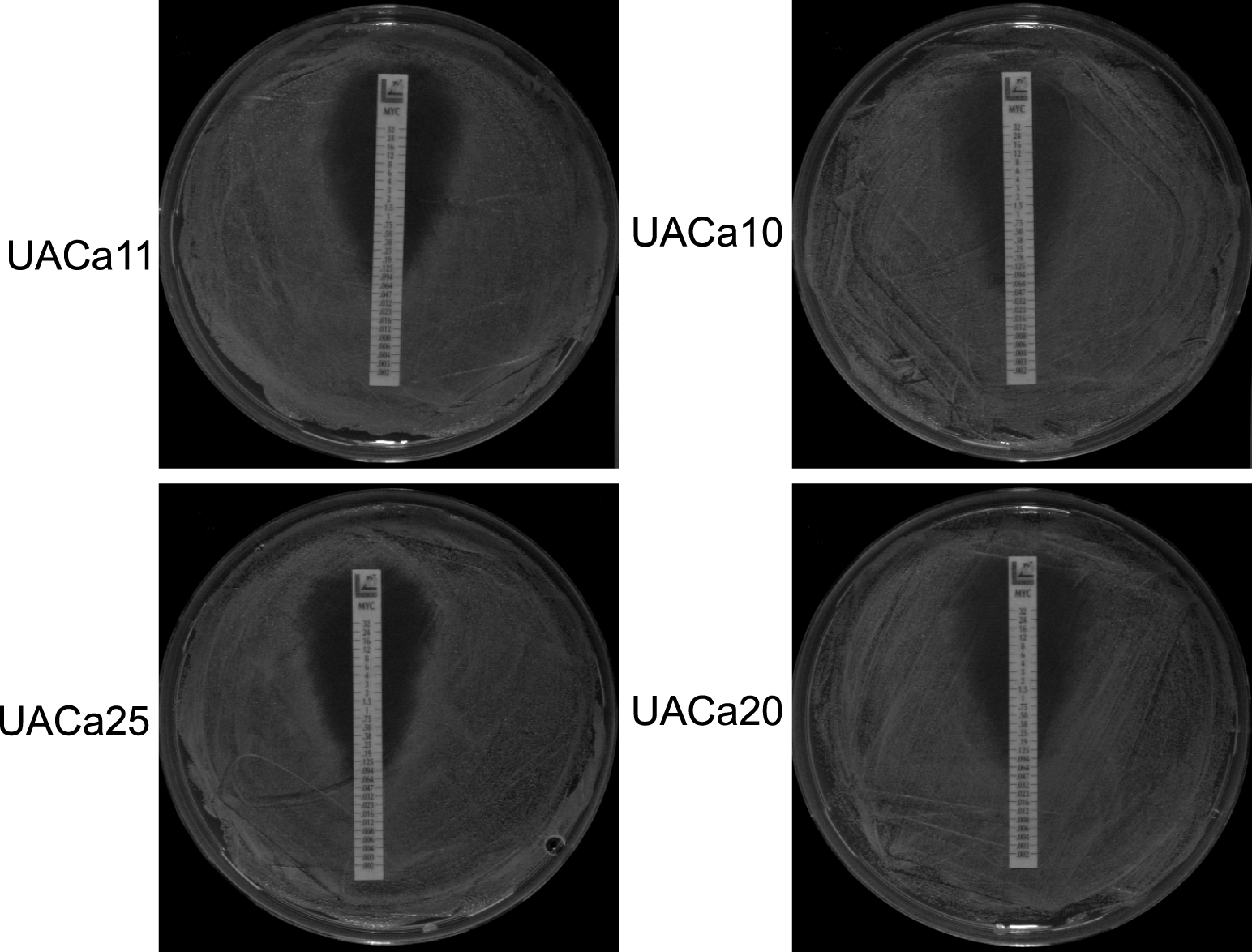

Supplement: S2 Fig — UACa11 and UACa25 had clear zones of inhibition and the MIC was 0.094 mg/L for both. UACa10 and UACa20 did not show a clear zone of inhibition, but a zone with reduced growth, the MIC90 was estimated to be 0.094 mg/L and 0.064 mg/L, respectively. (TIF) [file ppat.1012076.s010.tif]

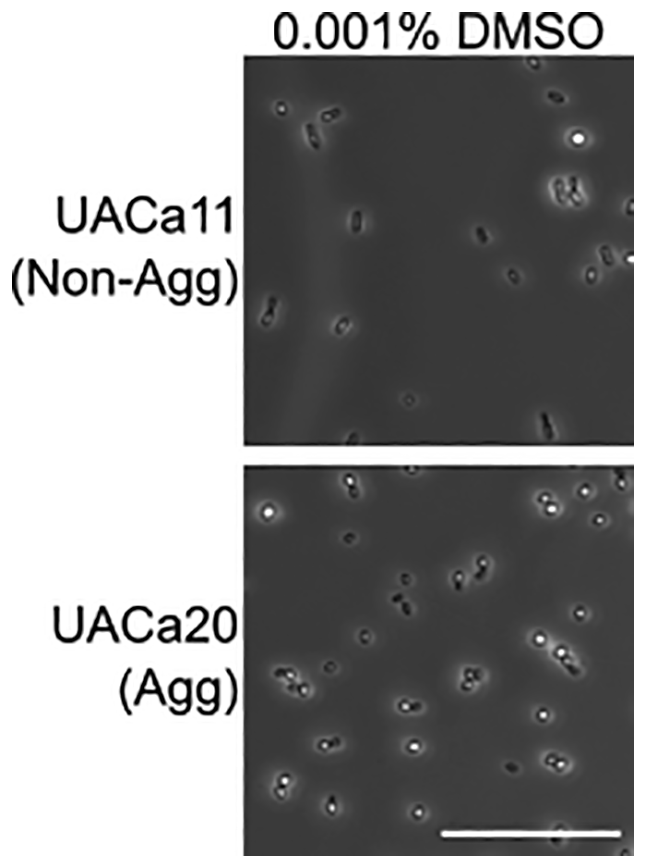

Supplement: S3 Fig — UACa11 and UACa20 grown in RPMI-1640 with 0.001% DMSO as a control to ensure that the antifungal carrier does not cause growth defects. Scale bar represents 50 μm. (TIF) [file ppat.1012076.s011.tif]

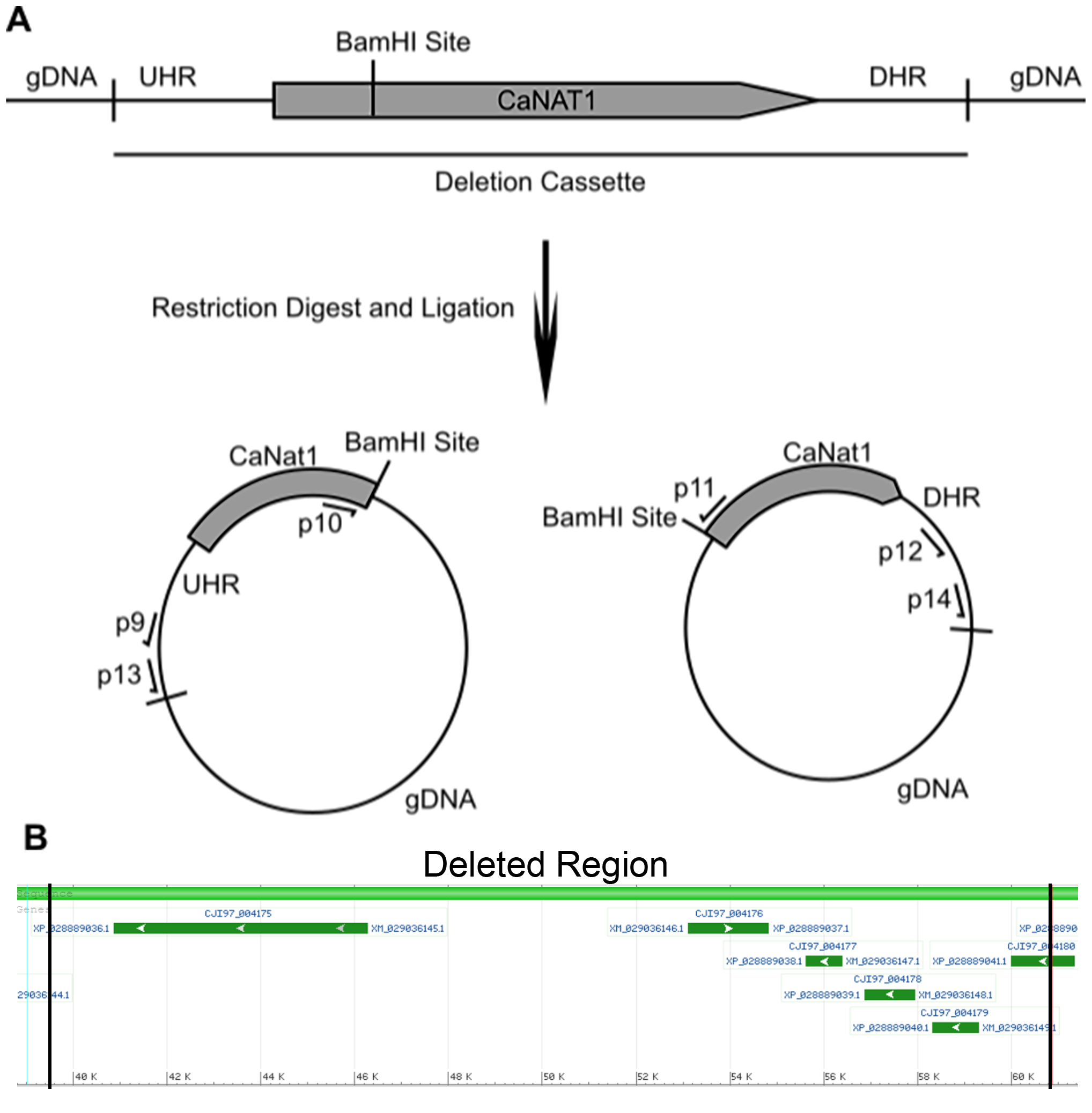

Supplement: S4 Fig — (A) Genomic DNA from the als4112Δ mutant was digested with BamHI to break apart the CaNat1 marker locus and fragment the genomic DNA, ligation followed to form circular DNA that could be amplified with PCR. Primers designed to amplify parts of the CaNat1 and the upstream homology region (UHR) or CaNat1 and the downstream homology region (DHR) of the deletion cassette were used to generate PCR products which were subjected toSanger sequencing. (B) Black lines indicate the boundaries of the deletion cassette as determined by BLAST searches of the sequences from the inverse PCR against the B11221 genome assembly on NCBI. (TIF) [file ppat.1012076.s012.tif]

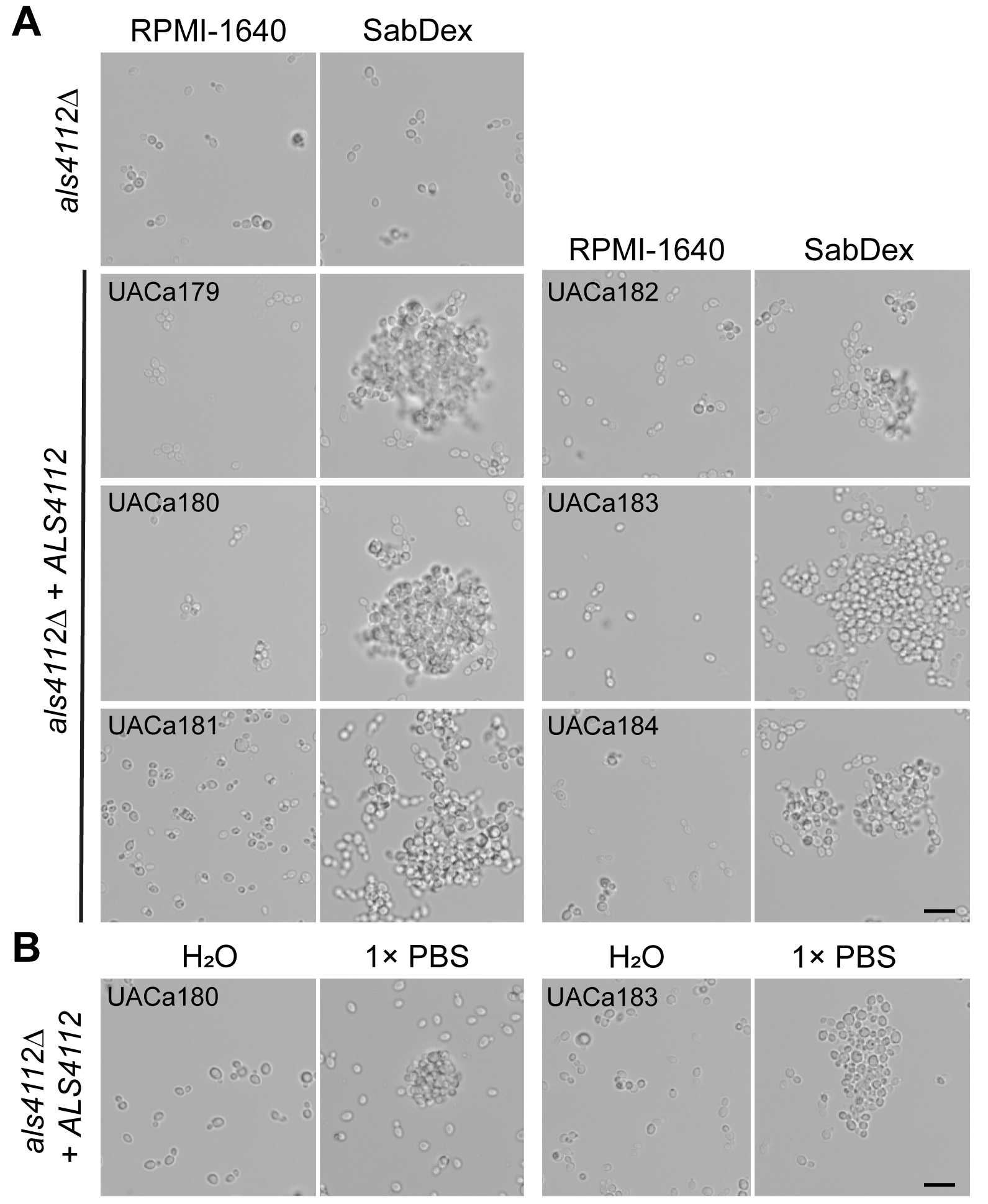

Supplement: S5 Fig — (A) Media-induced aggregation is abolished in the als4112Δ strain (UACa177) but can be restored by complementation with a randomly inserting construct harbouring ALS4112. Light microscopic (brightfield) images of cells of the indicated strains (UACa177, UACa179-184) in the indicated medium (top). Cells grown in RPMI-1640 displayed no aggregation. Only in strains with the ALS4112-construct, aggregation could be induced by growth in SabDex. (B) Aggregation of als4112Δ complemented with ALS4112 is lost when media is replaced with water but is retained when replaced with 1× PBS. Light microscopy (brightfield) of cells grown overnight in SabDex followed by either replacement of media with ddH2O or 1× PBS. Scale bars represent 10 μm. (TIF) [file ppat.1012076.s013.tif]
